# 1-Isopropyl-4-nitro-6-meth­oxy-1*H*-benzimidazole

**DOI:** 10.1107/S160053680801859X

**Published:** 2008-06-25

**Authors:** Michael D. Moore, Prashi Jain, Patrick T. Flaherty, Peter L. D. Wildfong

**Affiliations:** a Mylan School of Pharmacy, Duquesne University, 600 Forbes Avenue, Pittsburgh, PA 15282, USA

## Abstract

There are two independent mol­ecules in the asymmetric unit of the title compound, C_11_H_13_N_3_O_3_. The inter­planar angles for the two rings of the benzimidazole ring system is 2.21 (12)° in one mol­ecule and 0.72 (12)° in the other. The nitro group is twisted in the same direction relative to the least-squares plane through its attached benzene ring in both mol­ecules, with inter­planar angles of 15.22 (9) and 18.02 (8)°. In the crystal structure, mol­ecules are stacked along the *a* axis through π–π inter­actions (centroid–centroid distance 4.1954 Å). C—H⋯O hydrogen bonds are also present.

## Related literature

For background to the biological applications of benzimidazole cores, see: Townsend & Revankar (1970 ▶); Kamal *et al.* (2006 ▶); Bentancor *et al.* (2004 ▶); Somsak *et al.* (2003 ▶); Scholz *et al.* (2003 ▶); Sachs *et al.* (1995 ▶); Shin *et al.* (1997 ▶); Chackalamannil *et al.* (2003 ▶); Nicolaou *et al.* (1998 ▶); Lanusse & Prichard (1993 ▶); Wang (1984 ▶); Banks (1984 ▶); Sharma & Abuzar (1983 ▶); Lopez-Rodriguez *et al.* (2002 ▶). For other related literature on benzimidazole cores, see: Elderfield *et al.* (1946 ▶); Grimmett (2002 ▶); Kumar *et al.* (1982 ▶); Mizzoni & Spoerri (1945 ▶); Reddy & Reddy (1979 ▶). For related literature, see: Flaherty *et al.* (2008 ▶).
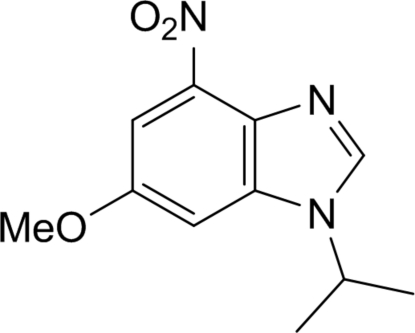


## Experimental

### 

#### Crystal data

C_11_H_13_N_3_O_3_
*M* *_r_* = 235.24
Orthorhombic, 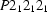

*a* = 7.63920 (10) Å
*b* = 16.0029 (3) Å
*c* = 17.9496 (3) Å
*V* = 2194.33 (6) Å^3^
*Z* = 8
Mo *K*α radiation
μ = 0.11 mm^−1^
*T* = 150 (2) K
0.43 × 0.33 × 0.28 mm

#### Data collection

Bruker SMART APEXII diffractometer
Absorption correction: multi-scan (*SADABS*; Sheldrick, 2002 ▶) *T*_min_ = 0.959, *T*_max_ = 0.971
39389 measured reflections
3989 independent reflections
3649 reflections with *I* > 2σ(*I*)
*R*_int_ = 0.026

#### Refinement

*R*[*F* ^2^ > 2σ(*F* ^2^)] = 0.034
*wR*(*F* ^2^) = 0.099
*S* = 1.09
3989 reflections
313 parameters
H-atom parameters constrained
Δρ_max_ = 0.47 e Å^−3^
Δρ_min_ = −0.20 e Å^−3^

### 

Data collection: *APEX2* and *SMART* (Bruker, 1998 ▶); cell refinement: *SAINT* (Bruker, 1998 ▶); data reduction: *SAINT*; program(s) used to solve structure: *SHELXS97* (Sheldrick, 2008 ▶); program(s) used to refine structure: *SHELXL97* (Sheldrick, 2008 ▶); molecular graphics: *ORTEP-3 for Windows* (Farrugia, 1997 ▶); software used to prepare material for publication: *SHELXTL* (Sheldrick, 2008 ▶) and *PLATON* (Spek, 2003 ▶).

## Supplementary Material

Crystal structure: contains datablocks I, global. DOI: 10.1107/S160053680801859X/zl2123sup1.cif

Structure factors: contains datablocks I. DOI: 10.1107/S160053680801859X/zl2123Isup2.hkl

Additional supplementary materials:  crystallographic information; 3D view; checkCIF report

## Figures and Tables

**Table 1:** Hydrogen-bond geometry (Å, °)

*D*—H⋯*A*	*D*—H	H⋯*A*	*D*⋯*A*	*D*—H⋯*A*
C15—H1⋯O6^i^	0.93	2.46	3.3670 (8)	164
C4—H15⋯O3^ii^	0.93	2.49	3.3723 (8)	159

Symmetry codes: (i) 
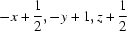
; (ii) 
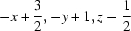
.

**Fig. 1.**
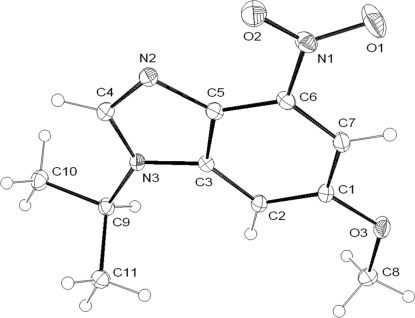
The molecular structure of the title compound, shown with 30% probability displacement ellipsoids (arbitrary spheres for H atoms).

**Fig. 2.**
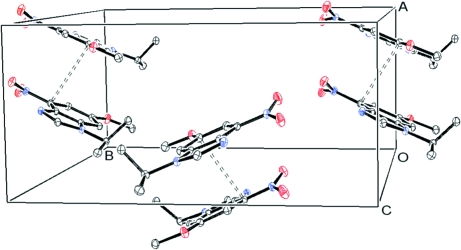
A packing diagram of (I) showing the π-π stacking interactions along the a axis between the benzimidazole rings (dashed line).

**Fig. 3.**
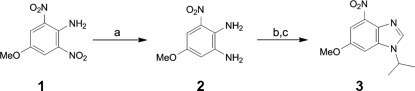
The molecular synthesis scheme used to produce the title compound (3). The following were also used: a: (NH4)2S,EtOH; b: formic acid, acetone, THF; c: conc. HCl, formic acid.

## supplementary crystallographic information

### Comment

The benzimidazole core has found application in multiple biologically active compounds including ATP mimics (Townsend & Revankar, 1970, Kamal *et al.*, 2006, Bentancor *et al.*, 2004, Somsak *et al.*, 2003, Scholz *et al.*, 2003), ATPase inhibitors (Sachs *et al.*, 1995, Shin *et al.*, 1997), peptide mimics (Chackalamannil *et al.*, 2003, Nicolaou *et al.*, 1998, antihelmentics (Lanusse & Prichard, 1993, Wang, 1984, Banks, 1984, Sharma & Abuzar, 1983), and serotonergic compounds (Lopez-Rodriguez *et al.*, 2002). Although multiple strategies exist to synthesize the imidazole ring onto the parent phenyl ring, the most prevalent strategy utilizes an *ortho*-phenyldiamine precursor, for example, compound 2 (Grimmett, 2002). Subsequent *N*-alkylation on the benzimidazole core presents an ambident nucleophile (Kumar *et al.*, 1982, Reddy & Reddy, 1979). Alkylation on the preformed benzimidazole has been shown to occur on the most nucleophilic nitrogen. However, this strategy often presents a mixture of products and fails completely with bulky, less-reactive electrophiles. A strategy of one-pot *N*-alkylation on the precursor phenyldiamine has been developed, followed by cyclization to afford 1-*N*-alkyl-4-nitro-6-methoxybenzimidazole (Flaherty *et al.*, In preparation). Unequivocal characterization of the alkylation site was essential to determine the utility of this method.

There are two molecules in the asymmetric unit of the title compound (Fig. 1). The nitro groups are twisted away from the least squares plane of the attached benzene rings, with the O2—N1—C6—C5 torsion angles being 17.24 (2)° and 14.72 (2)° for each molecule in the asymmetric unit, respectively, and the O1—N1—C6—C7 torsion angles being 18.51 (2)° and 15.10 (2)° for each molecule, respectively. Molecules in the crystal structure (Fig. 2) are stacked in parallel pairs along the *a* axis through π-π interactions, where the distance between C1 to C6 (centroid *Cg*2) and C12 to C18 (centroid *Cg*5) is 4.1954 Å [symmetry operation: *x*, *y*, *z*]. The crystal structure is further stabilized by weak C—H···O hydrogen bonds (Table 1).  Additional stabilization arises from C—H···π interactions involving C22—H10A with C3—C4—C5—N2—N3 (centroid *Cg*1) and C10—H14C with C14—C15—C16—N5—N6 (centroid *Cg*4) imidazole rings having X—Cg···H distances of 3.6317 (17) Å and 3.6818 (17) Å, respectively.

### Experimental

Known compound 1 (Fig. 3) could be prepared in one step from commercially available starting material according to the procedure of Elderfield (Elderfield *et al.*, 1946). Desymmetrization by Zinin mono reduction (Mizzoni & Spoerri, 1945) to 5-methoxy-3-nitrobenzene-1,2-diamine, 2 (Fig. 3), proceeded well. Reductive alkylation between 2 (Fig.3) and acetone generated the 2-isopropyl intermediate which was directly cyclized to 3 (Fig. 3) in formic acid and concentrated hydrochloric acid.

All compounds were obtained from Aldrich or Acros and used as supplied unless otherwise indicated. All reactions were conducted under an atmosphere of N_2_ unless otherwise indicated. Melting points were determined on a MelTemp apparatus and are uncorrected. ^1^H NMR analyses were determined on a 400 MHz Brucker NMR, and elemental analyses were preformed by Atlantic Microlabs.

CAUTION: Although the nitration to generate 1 (Fig. 3) proceeded in a controlled manner consistent with the literature (Elderfield *et al.*, 1946) and the differential scanning calorimetry of solid 1 (Fig. 3) did not indicate an exothermic event upon melting, these compounds should be handled with great care and proper precaution.

5-Methoxy-3-nitrobenzene-1,2-diamine (2, Fig. 3): A solution of 4-methoxy-2,6-dinitroaniline (Elderfield *et al.*, 1946) (1, Fig.3) (1.07 g, 5.0 mmole) in 50 ml of 100% EtOH was added to a 10% aqueous solution of (NH_4_)_2_S. This mixture was warmed to 333 K (60 °C). and stirred at 333 K (60 °C) for 1.5 h. This mixture was cooled to 273 K (0 °C) and the solid was collected on a #1 Whatman filter paper. The collected solids were washed with 5 ml of ice-cold CS_2_ then 5.0 ml of ice cold water to afford 0.65 g red needles after drying (70%). MP: 456–457.9 K (183–184.9 °C). ^1^H NMR (CDCl_3_) δ 3.53 (br s, 2H), 3.78 (s, 3H), 5.70 (s, 3H), 6.63 (s, 1H), 7.17 (s, 1H). Anal. Calcd for C_7_H_9_N_3_O_3_: C, 45.90; H, 4.95; N, 22.94. Found: C, 45.97; H, 4.99; N, 22.82.

1-Isopropyl-6-methoxy-4-nitro-1*H*-benzo[*d*]imidazole (3, Fig. 3): A solution of NaHB(O_2_CH)_3_ was prepared by slowly adding 99% formic acid (10.0 ml, 290 mmole) to a suspension of NaBH_4_ (1.83 g, 48.4 mmole) in THF (50 ml) at 273 K (0 °C) followed by stirring for an additional 5 min at 273 K (0 °C). To this solution was added a solution of 5-methoxy-3-nitrobenzene-1,2-diamine (2, Fig. 3) (2.1609 g, 11.8 mmole) in ACS grade acetone (20 ml) and THF (50.0 ml). The ice bath was removed and the mixture was permitted to stir for an additional 2 h at 296 K (23 °C). The solvent was removed under reduced pressure and the residue was dissolved into ice-cold 200 ml formic acid and then 60.0 ml concentrated HCl was added at 273 K (0 °C). This was brought directly to reflux for 15 min. The reaction was cooled to ambient temperature and the solvent was removed under reduced pressure. The residue was taken up into 15 ml ice-cold water and with cooling with an ice bath was made basic with a slight excess of 6 N NaOH. The precipitated dark solid was collected on #1 Whatman filter paper to 2.8 g. This solid was separated on SiO_2_ with 1:1 hexanes/ethyl acetate to afford 1.7 g of a bright yellow solid (74%). This material was recrystallized from CHCl_3_ to produce the material for crystallographic analysis. MP: 399–400 K (126–127 °C). ^1^H (CDCl_3_): δ 1.67 (2 t, 30 Hz, 6H), 3.96 (s, 1H), 4.69 (m, 1H), 7.24 (s, 1H), 7.82 (s, 1H), 8.26 (s, 1H). Anal. Calcd for C_11_H_13_N_3_O_3_: C, 56.16; H, 5.57; N, 17.86. C, 56.45; H,5.62; N, 17.55.

### Refinement

H atoms were positioned geometrically (aromatic C—H = 0.93 Å, methyl C—H = 0.96 Å, and methylene C–H = 0.98 Å) and treated with a riding model in subsequent refinement cycles. The isotropic displacement parameters were set to 1.2 *U*_eq_ (C) or 1.5 *U*_eq_ (methyl C) of the carrier atom.

### Crystal data

**Table:**

C_11_H_13_N_3_O_3_	*F*_000_ = 992.0
*M**_r_* = 235.24	*D*_x_ = 1.424 Mg m^−^^3^
Orthorhombic, *P*2_1_2_1_2_1_	Mo *K*α radiation λ = 0.71073 Å
Hall symbol: P 2ac 2ab	Cell parameters from 12882 reflections
*a* = 7.63920 (10) Å	θ = 2.3–30.9º
*b* = 16.0029 (3) Å	µ = 0.11 mm^−^^1^
*c* = 17.9496 (3) Å	*T* = 150 (2) K
*V* = 2194.33 (6) Å^3^	Rhomboid, yellow
*Z* = 8	0.43 × 0.33 × 0.28 mm

### Data collection

**Table:**

Bruker SMART APEXII diffractometer	3989 independent reflections
Radiation source: fine-focus sealed tube	3649 reflections with *I* > 2σ(*I*)
Monochromator: graphite	*R*_int_ = 0.026
*T* = 296(2) K	θ_max_ = 31.2º
φ and ω scans	θ_min_ = 1.7º
Absorption correction: multi-scan(SADABS; Sheldrick, 2002)	*h* = −11→11
*T*_min_ = 0.959, *T*_max_ = 0.971	*k* = −23→23
39389 measured reflections	*l* = −25→26

### Refinement

**Table:**

Refinement on *F*^2^	Secondary atom site location: difference Fourier map
Least-squares matrix: full	Hydrogen site location: inferred from neighbouring sites
*R*[*F*^2^ > 2σ(*F*^2^)] = 0.034	H-atom parameters constrained
*wR*(*F*^2^) = 0.099	*w* = 1/[σ^2^(*F*_o_^2^) + (0.0626*P*)^2^ + 0.1952*P*] where *P* = (*F*_o_^2^ + 2*F*_c_^2^)/3
*S* = 1.09	(Δ/σ)_max_ = 0.006
3989 reflections	Δρ_max_ = 0.47 e Å^−^^3^
313 parameters	Δρ_min_ = −0.20 e Å^−^^3^
Primary atom site location: structure-invariant direct methods	Extinction correction: none

### Special details

**Table:**

Geometry. All e.s.d.'s (except the e.s.d. in the dihedral angle between two l.s. planes) are estimated using the full covariance matrix. The cell e.s.d.'s are taken into account individually in the estimation of e.s.d.'s in distances, angles and torsion angles; correlations between e.s.d.'s in cell parameters are only used when they are defined by crystal symmetry. An approximate (isotropic) treatment of cell e.s.d.'s is used for estimating e.s.d.'s involving l.s. planes.
Refinement. Refinement of *F*^2^ against ALL reflections. The weighted *R*-factor *wR* and goodness of fit *S* are based on *F*^2^, conventional *R*-factors *R* are based on *F*, with *F* set to zero for negative *F*^2^. The threshold expression of *F*^2^ > σ(*F*^2^) is used only for calculating *R*-factors(gt) *etc*. and is not relevant to the choice of reflections for refinement. *R*-factors based on *F*^2^ are statistically about twice as large as those based on *F*, and *R*- factors based on ALL data will be even larger.

### Fractional atomic coordinates and isotropic or equivalent isotropic displacement parameters (Å^2^)

**Table:**

	*x*	*y*	*z*	*U*_iso_*/*U*_eq_	
O6	0.21889 (17)	0.44035 (7)	0.15295 (6)	0.0298 (2)	
N4	0.04946 (18)	0.68089 (7)	0.29007 (7)	0.0257 (2)	
C14	0.24765 (18)	0.47797 (8)	0.35375 (7)	0.0192 (2)	
N6	0.28545 (16)	0.45465 (7)	0.42604 (6)	0.0205 (2)	
C16	0.17746 (18)	0.55944 (8)	0.35923 (7)	0.0201 (2)	
C18	0.13756 (19)	0.55466 (9)	0.22533 (8)	0.0233 (3)	
H5	0.0985	0.5799	0.1817	0.028*	
C13	0.26894 (18)	0.43410 (8)	0.28697 (7)	0.0209 (2)	
H7	0.3191	0.3812	0.2856	0.025*	
O5	−0.0279 (2)	0.70366 (8)	0.23365 (7)	0.0401 (3)	
C17	0.12331 (19)	0.59655 (8)	0.29232 (8)	0.0213 (2)	
C12	0.2109 (2)	0.47402 (9)	0.22273 (7)	0.0222 (3)	
N5	0.17167 (17)	0.58463 (7)	0.43318 (7)	0.0236 (2)	
O4	0.0687 (2)	0.72566 (7)	0.34467 (7)	0.0436 (3)	
C21	0.2167 (2)	0.30465 (8)	0.43829 (8)	0.0258 (3)	
H9A	0.1150	0.3176	0.4675	0.039*	
H9B	0.2632	0.2517	0.4537	0.039*	
H9C	0.1850	0.3020	0.3866	0.039*	
C19	0.2891 (2)	0.35769 (9)	0.14715 (8)	0.0290 (3)	
H11A	0.4060	0.3569	0.1668	0.043*	
H11B	0.2913	0.3410	0.0958	0.043*	
H11C	0.2172	0.3197	0.1750	0.043*	
C20	0.35454 (19)	0.37233 (8)	0.44953 (8)	0.0222 (2)	
H8	0.4568	0.3588	0.4189	0.027*	
C15	0.2357 (2)	0.52027 (8)	0.46970 (8)	0.0235 (3)	
H1	0.2463	0.5195	0.5213	0.028*	
C22	0.4111 (2)	0.37599 (10)	0.53068 (8)	0.0300 (3)	
H10A	0.4943	0.4204	0.5372	0.045*	
H10B	0.4640	0.3238	0.5445	0.045*	
H10C	0.3108	0.3861	0.5616	0.045*	
C5	0.63745 (18)	0.55518 (8)	0.10934 (7)	0.0200 (2)	
N1	0.51798 (17)	0.67741 (7)	0.17962 (7)	0.0257 (2)	
O3	0.74288 (17)	0.44725 (7)	0.31636 (5)	0.0290 (2)	
C6	0.60053 (18)	0.59506 (8)	0.17718 (8)	0.0209 (2)	
N3	0.74022 (16)	0.45062 (7)	0.04131 (6)	0.0197 (2)	
C3	0.71541 (17)	0.47535 (8)	0.11420 (7)	0.0185 (2)	
C1	0.71502 (19)	0.47734 (9)	0.24614 (7)	0.0215 (2)	
C2	0.75604 (18)	0.43486 (8)	0.18085 (7)	0.0205 (2)	
H18	0.8078	0.3823	0.1816	0.025*	
N2	0.61682 (17)	0.57845 (7)	0.03556 (6)	0.0233 (2)	
C7	0.63817 (19)	0.55723 (9)	0.24427 (8)	0.0229 (3)	
H20	0.6126	0.5847	0.2886	0.027*	
O1	0.5265 (2)	0.71814 (9)	0.23746 (8)	0.0493 (4)	
O2	0.44371 (17)	0.70267 (7)	0.12361 (7)	0.0342 (3)	
C11	0.6778 (2)	0.29985 (8)	0.03307 (8)	0.0252 (3)	
H13A	0.6454	0.3014	0.0847	0.038*	
H13B	0.7288	0.2465	0.0217	0.038*	
H13C	0.5757	0.3082	0.0028	0.038*	
C4	0.67924 (19)	0.51421 (8)	−0.00193 (8)	0.0231 (3)	
H15	0.6815	0.5125	−0.0537	0.028*	
C9	0.80998 (18)	0.36854 (8)	0.01721 (7)	0.0207 (2)	
H12	0.9166	0.3568	0.0457	0.025*	
C10	0.8569 (2)	0.37134 (10)	−0.06505 (8)	0.0268 (3)	
H14A	0.7523	0.3781	−0.0940	0.040*	
H14B	0.9138	0.3202	−0.0789	0.040*	
H14C	0.9343	0.4175	−0.0742	0.040*	
C8	0.8120 (2)	0.36434 (9)	0.32175 (8)	0.0270 (3)	
H22A	0.7336	0.3259	0.2978	0.041*	
H22B	0.8245	0.3494	0.3733	0.041*	
H22C	0.9242	0.3621	0.2978	0.041*	

### Atomic displacement parameters (Å^2^)

**Table:**

	*U*^11^	*U*^22^	*U*^33^	*U*^12^	*U*^13^	*U*^23^
O6	0.0465 (6)	0.0262 (5)	0.0168 (4)	0.0047 (5)	0.0021 (4)	−0.0026 (4)
N4	0.0284 (6)	0.0188 (5)	0.0301 (6)	−0.0002 (5)	−0.0001 (5)	0.0018 (4)
C14	0.0220 (6)	0.0178 (5)	0.0179 (5)	−0.0014 (5)	0.0023 (4)	−0.0004 (4)
N6	0.0256 (5)	0.0189 (5)	0.0169 (5)	0.0000 (4)	0.0010 (4)	−0.0004 (4)
C16	0.0227 (5)	0.0177 (5)	0.0201 (5)	−0.0023 (5)	0.0020 (5)	−0.0006 (4)
C18	0.0282 (6)	0.0230 (6)	0.0187 (6)	−0.0020 (5)	0.0006 (5)	0.0012 (5)
C13	0.0250 (6)	0.0192 (5)	0.0185 (5)	−0.0004 (5)	0.0031 (5)	−0.0010 (4)
O5	0.0549 (8)	0.0277 (6)	0.0377 (7)	0.0083 (6)	−0.0124 (6)	0.0042 (5)
C17	0.0233 (6)	0.0171 (5)	0.0236 (6)	−0.0016 (5)	0.0014 (5)	0.0007 (5)
C12	0.0277 (6)	0.0225 (6)	0.0165 (5)	−0.0024 (5)	0.0026 (5)	−0.0004 (5)
N5	0.0296 (6)	0.0204 (5)	0.0207 (5)	−0.0006 (4)	0.0031 (4)	−0.0032 (4)
O4	0.0650 (9)	0.0257 (5)	0.0402 (7)	0.0130 (6)	−0.0125 (7)	−0.0092 (5)
C21	0.0304 (7)	0.0203 (6)	0.0266 (6)	−0.0002 (5)	0.0000 (5)	−0.0001 (5)
C19	0.0358 (8)	0.0259 (7)	0.0252 (6)	−0.0001 (6)	0.0027 (6)	−0.0064 (5)
C20	0.0237 (6)	0.0210 (6)	0.0218 (6)	0.0029 (5)	0.0009 (5)	0.0013 (5)
C15	0.0294 (7)	0.0218 (6)	0.0194 (6)	−0.0021 (5)	0.0019 (5)	−0.0031 (5)
C22	0.0319 (7)	0.0327 (7)	0.0254 (7)	0.0010 (6)	−0.0068 (6)	0.0043 (6)
C5	0.0221 (5)	0.0175 (5)	0.0204 (6)	−0.0012 (5)	−0.0016 (5)	−0.0002 (4)
N1	0.0285 (6)	0.0191 (5)	0.0296 (6)	0.0024 (4)	−0.0017 (5)	−0.0039 (5)
O3	0.0439 (6)	0.0263 (5)	0.0167 (4)	0.0065 (5)	−0.0040 (4)	0.0010 (4)
C6	0.0228 (6)	0.0167 (5)	0.0230 (6)	0.0002 (5)	−0.0020 (5)	−0.0028 (5)
N3	0.0256 (5)	0.0179 (5)	0.0157 (4)	0.0001 (4)	−0.0005 (4)	−0.0007 (4)
C3	0.0215 (5)	0.0170 (5)	0.0170 (5)	−0.0018 (5)	−0.0001 (4)	−0.0016 (4)
C1	0.0257 (6)	0.0225 (6)	0.0162 (5)	−0.0012 (5)	−0.0023 (5)	−0.0003 (5)
C2	0.0247 (6)	0.0187 (5)	0.0181 (5)	0.0000 (5)	−0.0021 (5)	0.0001 (4)
N2	0.0291 (6)	0.0197 (5)	0.0210 (5)	0.0005 (4)	−0.0031 (4)	0.0008 (4)
C7	0.0275 (6)	0.0226 (6)	0.0185 (5)	0.0001 (5)	−0.0022 (5)	−0.0032 (5)
O1	0.0723 (10)	0.0350 (7)	0.0405 (8)	0.0216 (7)	−0.0183 (7)	−0.0192 (6)
O2	0.0425 (7)	0.0281 (5)	0.0320 (6)	0.0112 (5)	−0.0047 (5)	0.0008 (4)
C11	0.0312 (7)	0.0197 (6)	0.0247 (6)	−0.0003 (5)	0.0022 (5)	−0.0016 (5)
C4	0.0292 (6)	0.0214 (6)	0.0187 (6)	−0.0005 (5)	−0.0022 (5)	0.0017 (5)
C9	0.0237 (6)	0.0195 (5)	0.0189 (5)	0.0024 (5)	0.0000 (5)	−0.0020 (4)
C10	0.0306 (7)	0.0297 (7)	0.0201 (6)	0.0021 (6)	0.0036 (5)	−0.0017 (5)
C8	0.0335 (7)	0.0232 (6)	0.0243 (6)	−0.0003 (6)	−0.0019 (6)	0.0051 (5)

### Geometric parameters (Å, °)

**Table:**

O6—C12	1.3649 (16)	C5—N2	1.3846 (17)
O6—C19	1.4312 (18)	C5—C6	1.4035 (18)
N4—O4	1.2229 (17)	C5—C3	1.4122 (18)
N4—O5	1.2279 (18)	N1—O2	1.2231 (17)
N4—C17	1.4635 (17)	N1—O1	1.2277 (17)
C14—N6	1.3807 (16)	N1—C6	1.4615 (17)
C14—C13	1.3986 (17)	O3—C1	1.3659 (16)
C14—C16	1.4131 (18)	O3—C8	1.4314 (17)
N6—C15	1.3643 (17)	C6—C7	1.3781 (19)
N6—C20	1.4805 (16)	N3—C4	1.3620 (17)
C16—N5	1.3879 (17)	N3—C3	1.3800 (16)
C16—C17	1.4022 (18)	N3—C9	1.4820 (16)
C18—C17	1.3810 (19)	C3—C2	1.3955 (17)
C18—C12	1.408 (2)	C1—C2	1.3907 (18)
C18—H5	0.9300	C1—C7	1.4073 (19)
C13—C12	1.3908 (18)	C2—H18	0.9300
C13—H7	0.9300	N2—C4	1.3179 (18)
N5—C15	1.3153 (18)	C7—H20	0.9300
C21—C20	1.524 (2)	C11—C9	1.5195 (19)
C21—H9A	0.9600	C11—H13A	0.9600
C21—H9B	0.9600	C11—H13B	0.9600
C21—H9C	0.9600	C11—H13C	0.9600
C19—H11A	0.9600	C4—H15	0.9300
C19—H11B	0.9600	C9—C10	1.5202 (19)
C19—H11C	0.9600	C9—H12	0.9800
C20—C22	1.520 (2)	C10—H14A	0.9600
C20—H8	0.9800	C10—H14B	0.9600
C15—H1	0.9300	C10—H14C	0.9600
C22—H10A	0.9600	C8—H22A	0.9600
C22—H10B	0.9600	C8—H22B	0.9600
C22—H10C	0.9600	C8—H22C	0.9600
			
C12—O6—C19	116.63 (11)	N2—C5—C6	133.21 (12)
O4—N4—O5	123.03 (13)	N2—C5—C3	110.51 (11)
O4—N4—C17	118.16 (12)	C6—C5—C3	116.26 (11)
O5—N4—C17	118.82 (13)	O2—N1—O1	122.99 (13)
N6—C14—C13	130.20 (12)	O2—N1—C6	118.27 (12)
N6—C14—C16	105.27 (11)	O1—N1—C6	118.75 (13)
C13—C14—C16	124.53 (12)	C1—O3—C8	116.52 (11)
C15—N6—C14	105.88 (11)	C7—C6—C5	121.09 (12)
C15—N6—C20	128.35 (11)	C7—C6—N1	117.39 (12)
C14—N6—C20	125.65 (11)	C5—C6—N1	121.51 (12)
N5—C16—C17	133.37 (12)	C4—N3—C3	106.19 (11)
N5—C16—C14	110.28 (11)	C4—N3—C9	128.25 (11)
C17—C16—C14	116.30 (11)	C3—N3—C9	125.47 (11)
C17—C18—C12	120.35 (12)	N3—C3—C2	130.47 (12)
C17—C18—H5	119.8	N3—C3—C5	105.00 (11)
C12—C18—H5	119.8	C2—C3—C5	124.53 (12)
C12—C13—C14	116.29 (12)	O3—C1—C2	124.77 (12)
C12—C13—H7	121.9	O3—C1—C7	114.03 (11)
C14—C13—H7	121.9	C2—C1—C7	121.20 (12)
C18—C17—C16	121.13 (12)	C1—C2—C3	116.45 (12)
C18—C17—N4	117.00 (12)	C1—C2—H18	121.8
C16—C17—N4	121.87 (12)	C3—C2—H18	121.8
O6—C12—C13	124.43 (13)	C4—N2—C5	103.73 (11)
O6—C12—C18	114.21 (12)	C6—C7—C1	120.47 (12)
C13—C12—C18	121.36 (12)	C6—C7—H20	119.8
C15—N5—C16	103.74 (11)	C1—C7—H20	119.8
C20—C21—H9A	109.5	C9—C11—H13A	109.5
C20—C21—H9B	109.5	C9—C11—H13B	109.5
H9A—C21—H9B	109.5	H13A—C11—H13B	109.5
C20—C21—H9C	109.5	C9—C11—H13C	109.5
H9A—C21—H9C	109.5	H13A—C11—H13C	109.5
H9B—C21—H9C	109.5	H13B—C11—H13C	109.5
O6—C19—H11A	109.5	N2—C4—N3	114.57 (12)
O6—C19—H11B	109.5	N2—C4—H15	122.7
H11A—C19—H11B	109.5	N3—C4—H15	122.7
O6—C19—H11C	109.5	N3—C9—C10	110.01 (11)
H11A—C19—H11C	109.5	N3—C9—C11	110.34 (11)
H11B—C19—H11C	109.5	C10—C9—C11	111.10 (12)
N6—C20—C22	109.87 (11)	N3—C9—H12	108.4
N6—C20—C21	110.38 (11)	C10—C9—H12	108.4
C22—C20—C21	110.53 (12)	C11—C9—H12	108.4
N6—C20—H8	108.7	C9—C10—H14A	109.5
C22—C20—H8	108.7	C9—C10—H14B	109.5
C21—C20—H8	108.7	H14A—C10—H14B	109.5
N5—C15—N6	114.83 (12)	C9—C10—H14C	109.5
N5—C15—H1	122.6	H14A—C10—H14C	109.5
N6—C15—H1	122.6	H14B—C10—H14C	109.5
C20—C22—H10A	109.5	O3—C8—H22A	109.5
C20—C22—H10B	109.5	O3—C8—H22B	109.5
H10A—C22—H10B	109.5	H22A—C8—H22B	109.5
C20—C22—H10C	109.5	O3—C8—H22C	109.5
H10A—C22—H10C	109.5	H22A—C8—H22C	109.5
H10B—C22—H10C	109.5	H22B—C8—H22C	109.5

### Hydrogen-bond geometry (Å, °)

**Table:**

*D*—H···*A*	*D*—H	H···*A*	*D*···*A*	*D*—H···*A*
C15—H1···O6^i^	0.93	2.46	3.3670 (8)	164
C4—H15···O3^ii^	0.93	2.49	3.3723 (8)	159

Symmetry codes: (i) −*x*+1/2, −*y*+1, *z*+1/2; (ii) −*x*+3/2, −*y*+1, *z*−1/2.
